# Cabozantinib exposure–response analysis for the phase 3 CheckMate 9ER trial of nivolumab plus cabozantinib versus sunitinib in first-line advanced renal cell carcinoma

**DOI:** 10.1007/s00280-022-04500-9

**Published:** 2023-01-10

**Authors:** Benjamin Duy Tran, Jing Li, Neang Ly, Raffaella Faggioni, Lorin Roskos

**Affiliations:** Exelixis, Inc., 1851 Harbor Bay Parkway, Alameda, CA USA

**Keywords:** Cabozantinib, Renal cell carcinoma, Nivolumab, Exposure–response analysis

## Abstract

**Purpose:**

In the phase 3 CheckMate 9ER trial, intravenous nivolumab (240 mg every 2 weeks) plus oral cabozantinib (40 mg/day) improved progression-free survival (PFS) versus sunitinib as first-line therapy for advanced renal cell carcinoma (RCC). To support cabozantinib dosing with the combination, this exposure–response analysis characterized the relationship of cabozantinib exposure with clinical endpoints.

**Methods:**

Dose modification was allowed with cabozantinib (holds and reductions) to manage adverse events (AEs). The population pharmacokinetics analysis was updated and used to generate individual predicted cabozantinib exposure measures. Kaplan–Meier plots and time-to-event Cox proportional hazard (CPH) exposure–response models characterized the relationship of cabozantinib exposure with PFS, dose modifications, and selected AEs.

**Results:**

Kaplan–Meier plots showed no clear difference in PFS across cabozantinib exposure quartiles. Cabozantinib exposure did not significantly affect the hazard of PFS in the CPH base model nor in the final model. In contrast, baseline albumin and nivolumab clearance had a significant effect on PFS. There was no significant relationship between cabozantinib clearance and risk of dose modification, but a significant relationship was identified between cabozantinib exposure and Grade ≥ 1 palmar-plantar-erythrodysesthesia and Grade ≥ 3 diarrhea in the exposure–response analysis.

**Conclusion:**

To optimize individual cabozantinib exposure, these data support the dose modification strategies in CheckMate 9ER for cabozantinib in patients with advanced RCC when combined with nivolumab.

**Supplementary Information:**

The online version contains supplementary material available at 10.1007/s00280-022-04500-9.

## Introduction

Over the last decade, improved understanding of the angiogenic and immunogenic biology of renal cell carcinoma (RCC) has led to development of tyrosine kinase inhibitors (TKI) that target the vascular endothelial growth factor receptor (VEGFR) and immune checkpoint inhibitors that target the programmed cell death 1 (PD-1) and cytotoxic T lymphocyte antigen 4 (CTLA-4) receptors [1, 2]. Cabozantinib is a TKI with multiple targets implicated in tumor pathology, including tumor growth, metastasis, and angiogenesis [3]. These include VEGFR, MET, and the TAM family of receptor kinases (TYRO3, AXL, and MER). Cabozantinib also has immunomodulatory properties that may mitigate tumor-induced immunosuppression and enhance response to ICIs [4, 5].

The cabozantinib tablet formulation (Cabometyx^™^) is approved at a 60-mg once daily (QD) dose as a single-agent therapy for patients with advanced RCC in the USA [6], the European Union (EU) [7], and Japan [8]. Because of high interpatient variability in cabozantinib clearance, patients receiving cabozantinib may require dose modification to optimize exposure and improve tolerability while maintaining efficacy [9]. Patients with low clearance and high exposure to cabozantinib are more likely to require dose modification to manage adverse events (AEs), but this is not predicted to adversely affect efficacy in exposure–response analyses [9].

Given its immunomodulatory activity, cabozantinib is being evaluated in combination with immune checkpoint inhibitors as part of its clinical development. The combination of cabozantinib with nivolumab (a PD-1 immune checkpoint inhibitor antibody) showed promising preliminary antitumor activity in a phase 1 study in patients with advanced genitourinary malignancies, providing a rationale for the combination [10].

In the phase 3 CheckMate 9ER trial (NCT03141177), nivolumab plus cabozantinib significantly improved progression-free survival (PFS) compared with sunitinib (hazard ratio [HR] 0.51, 95% confidence interval [CI] 0.41–0.64; *p* < 0.001) [11]. Nivolumab plus cabozantinib was generally well tolerated with dose modification. Dose holds and reductions were allowed for cabozantinib, and dose holds were allowed for nivolumab. Fifty-six percent of patients required a dose reduction of cabozantinib, but the rate of discontinuations due to AEs was low (5.6% of patients discontinued both nivolumab and cabozantinib due to AEs), indicating effective AE management with dose modification to maintain tolerability. This combination has been approved in the USA, the EU, and Japan as a first-line treatment for patients with advanced RCC [7, 8, 12].

Here, we describe a cabozantinib exposure–response analysis based on efficacy and safety data from patients with RCC administered nivolumab plus cabozantinib in CheckMate 9ER. A population pharmacokinetics (PopPK) analysis of cabozantinib was updated and used to generate individual predicted cabozantinib exposure measures for the exposure–response analyses. The objective was to characterize the relationship of cabozantinib exposure with efficacy and safety endpoints and to investigate the effects of selected covariates on the exposure–response relationship of cabozantinib with efficacy.

## Materials and methods

### Study design and data

The exposure–response analyses were conducted using data from the phase 3 CheckMate 9ER trial of nivolumab plus cabozantinib versus sunitinib in first-line advanced RCC. CheckMate 9ER was an open-label, randomized phase 3 trial of nivolumab (240 mg intravenously every 2 weeks) plus cabozantinib (40 mg orally QD) versus sunitinib (50 mg orally QD for 4 weeks on treatment, then 2 weeks off) in patients with previously untreated advanced or metastatic RCC [11, 13]. The primary endpoint was PFS per Response Evaluation Criteria in Solid Tumors (RECIST) version 1.1 by blinded independent central review (BICR). Randomization was stratified by International Metastatic RCC Database Consortium (IMDC) risk score, tumor programmed death-ligand 1 (PD-L1) expression, and geographic region. Patients were ≥ 18 years of age with a clear-cell RCC histology and measurable disease per RECIST v1.1.

A total of 651 patients were randomized to receive nivolumab plus cabozantinib (*n* = 323) or sunitinib (*n* = 328) until disease progression or unacceptable toxicity, with a maximum duration of 2 years for nivolumab treatment. Dose holds to manage AEs were permitted for both cabozantinib and nivolumab. Dose reductions to manage AEs were only permitted for cabozantinib: from 40 to 20 mg QD, and then to 20 mg every other day (QOD).

Radiographic assessments were performed at screening, Week 12, then every 6 weeks until Week 60, then every 12 weeks until progression. Safety was assessed every 2 weeks. Blood samples for PK assessment of cabozantinib were collected in Cycles 1, 3, 4 and 7 (cycle = 2 weeks for nivolumab), at the same time as pre-dose PK samples for nivolumab, provided the time of the draw was approximately ≥ 8 h after the previous evening dose of cabozantinib to ensure sampling occurred after the T_max_ of roughly 3–4 h [14]. Plasma concentration analyses for cabozantinib were performed by a validated liquid chromatography– tandem mass spectrometry assay (lower limit of quantitation was 0.5 ng/mL).

### Population pharmacokinetic model

The PopPK analysis included plasma cabozantinib concentration–time data from CheckMate 9ER and 10 studies from a previously developed integrated PopPK model that characterized cabozantinib concentration data from healthy subjects and patients with various types of malignancies [15, 16]. Protocols for all studies were approved by institutional review boards of participating institutions and written informed consent was obtained from patients prior to enrollment.

The analytical data preparation has been previously described [15]. The previous PopPK model comprised a 2-compartment model with first-order elimination and dual-absorption (first-order + zero-order) processes [16]. That model was used as the base model for the current analysis. The impact of previously evaluated demographic covariates and cancer type on cabozantinib apparent clearance (CL/F) was re-evaluated in the updated dataset, which included data from CheckMate 9ER patients. An additional covariate effect related to combination therapy with nivolumab was also assessed. The final PK model was used to generate individual predicted cabozantinib exposures using dosing history from patients in the CheckMate 9ER trial for the exposure–response analyses.

### Exposure–response analysis for time-to-event endpoints

#### Efficacy and safety endpoints

Time-to-event analyses were performed to characterize the exposure–response relationship between cabozantinib exposure and PFS (defined as the time from randomization to radiographic progression per RECIST v1.1 by BICR, or death), cabozantinib dose modification, and each of the following AEs commonly associated with cabozantinib dose holds and reductions: palmar-plantar erythrodysesthesia (PPE; Grade ≥ 1), diarrhea (Grade ≥ 3), hypertension (Grade ≥ 3 [systolic BP > 160 mmHg or diastolic BP > 100 mmHg]), fatigue/asthenia (Grade ≥ 3), and alanine aminotransferase/aspartate aminotransferase (ALT/AST) elevation (Grade ≥ 3). An intended time-to-event analysis assessing the relationship of OS with cabozantinib exposure was not performed as there were too few events and limited follow-up at the time of the exposure–response analysis.

#### Dataset construction and preliminary analyses

Individual average cabozantinib concentrations from time zero to the event or censoring time (CAVG0T) were calculated from the estimated individual PK parameters of the final PopPK model and individual dosing history; CAVG0T was used for the exposure–response analysis dataset to link clinical endpoints with individual predicted cabozantinib exposure.

The average cabozantinib concentration, CAVG0T, in units of ng/mL was defined as (Eq. 1):

1 $${{CAVGOT}} = \frac{{{\text{AUC}}_{0 \ to \ t} }}{t \cdot 24}$$

where *t* represents time in days, and AUC is the area under the concentration–time curve (concentration × time in hours). CAVG0T is a time-invariant exposure metric. For the cabozantinib dose-modification endpoint, individual predicted apparent clearance was used to represent cabozantinib exposure. Patients who had at least one documented cabozantinib dose without any measurable PK concentration were assigned population-level PK parameter estimates for prediction of exposure.

Kaplan–Meier (KM) plots were constructed for each efficacy and safety endpoint prior to model development. Patients were grouped into quartiles based on CAVG0T or apparent clearance for corresponding clinical endpoints. The preliminary analyses were performed to identify potential relationships between cabozantinib exposure and each clinical endpoint. Only time to the first event was considered for the analyses of safety endpoints.

#### Cox proportional hazards (CPH) model

CPH models were used to describe the relative hazard for each of the clinical efficacy and safety endpoints.

The general form of the CPH model is represented by the equation (Eq. 2):

2 $$h\,(t,X)\, = \,h_{o} (t) \cdot {\text{exp}}\,(\beta \cdot X)$$

where *h (t*, *X)* denotes the hazard at time *t*, *h*_*o*_*(t)* is the background hazard function, *β* is a vector of the regression coefficients, and *X* is a matrix of covariates.

#### Model development

Separate CPH models were developed for each of the clinical endpoints using data from the CheckMate 9ER trial. The impact of cabozantinib exposure on relative hazard was evaluated during base model development. A drop in − 2 log likelihood (− 2LL) when including cabozantinib exposure in the CPH model was considered significant. Both linear (Eq. 3) and nonlinear (Eq. 4) functional forms were evaluated:

3 $$h\,(t,\,X_{ex} )\, = \,h_{o} (t) \cdot \exp \,(\beta_{ex1} \cdot X_{ex} )$$

4 $$h(t, {{ X}^{\mathrm{^{\prime}}}}_{ex})={h}_{o}\left(t\right)\cdot \mathit{exp}\left({\beta }_{ex2} \cdot {{X}^{\mathrm{^{\prime}}}}_{ex}\right)\,where\  {{X}^{\mathrm{^{\prime}}}}_{ex}= \frac{{X}_{ex}}{{X}_{ex}+{EC}_{50}}$$

where *X*_*ex*_ is the cabozantinib exposure (e.g, CAVG0T), *β*_*ex1*_ represents the slope in the log-linear model, *β*_*ex2*_ represents the maximum drug effect in the E_max_ model, and EC_50_ represents cabozantinib exposure at which half of the maximal effect is achieved.

#### Covariate analysis

Covariate effects were assessed for the PFS model. Covariates were added to the base model simultaneously to form a full model followed by stepwise backward elimination to identify the most parsimonious model. Statistical significance of covariate-parameter relationships was assessed with the Wald test, at *α* = 0.1 for inclusion. At each step of the backward elimination procedure, the least significant covariate–parameter relationship was eliminated, and the procedure was repeated until all covariates–parameters met the inclusion criteria.

Covariates evaluated were based on clinical judgment and mechanistic plausibility and included: age, baseline PD-L1 + tumor expression (≥ 1% vs. < 1% or indeterminate), baseline IMDC score (0 vs. 1–2 vs. 3–6), baseline Karnofsky performance status (≥ 90 vs. < 90), prior adjuvant or neo-adjuvant therapy for localized or locally advanced RCC (Yes vs. No), prior nephrectomy (Yes vs. No), prior radiotherapy (Yes vs. No), baseline lactate dehydrogenase (LDH) level (≤ 1.5 × upper limit of normal [ULN] vs. > 1.5 × ULN), time from initial disease diagnosis to randomization (< 1 year vs. ≥ 1 year), sex, baseline body weight, tumor burden (sum of the diameter of target lesions at baseline, > median vs. ≤ median), baseline albumin (5th percentile vs. median, 95th percentile vs. median), baseline nivolumab clearance (5th percentile vs. median, 95th percentile vs. median), and liver metastasis (Yes vs. No).

#### Final model evaluation and simulations

The predictive performance of the PopPK model and time-to-event models was evaluated using visual predictive checks (VPC). Simulations were also performed using the final CPH model parameter estimates to predict the incidence of efficacy and safety endpoints for different dose levels.

### Software

For PopPK modeling, analyses were performed using nonlinear mixed effects modeling methodology as implemented in the NONMEM software system, version 7.3 (ICON Development Solutions, Ellicott City, MD). For exposure–response analysis, time-to-event analyses were performed using the Cox proportional hazards regression (PHREG) procedure within SAS (version 9.4). Graphical analysis of the data or output from the models was performed using R software (version 3.6.1).

## Results

### CheckMate9ER pharmacokinetic analysis and population pharmacokinetic model

Of the 323 patients randomized to nivolumab plus cabozantinib in CheckMate 9ER, 320 received at least one dose of study treatment and 308 had at least one measurable cabozantinib concentration. Table 1 summarizes baseline demographics and covariates for the 320 patients.

**Table 1:** Summary of demographic and covariate data in patients who were randomized to cabozantinib plus nivolumab and received at least one dose of study treatment

Covariate	Summary statistic
Age (yr)	
*N*	320
Mean (SD)	61.4 (10.2)
Median (range)	62 (29─90)
Baseline body weight (kg)	
*N*	320
Mean (SD)	81.5 (18.0)
Median (range)	80.3 (36.0─159.3)
Baseline nivolumab clearance (L/h)	
*N*	315
Mean (SD)	0.009 (0.003)
Median (range)	0.009 (0.004─0.032)
Baseline albumin (g/L)	
*N*	309
Mean (SD)	40.4 (5.47)
Median (range)	41.0 (16.0─53.4)
Tumor burden^a^ (mm)	
*N*	290
Mean (SD)	85.6 (63.2)
Median (range)	69 (10─330)
Sex, *n* (%)	
Male	247 (77.2)
Female	73 (22.8)
Baseline Karnofsky performance status, *n* (%)	
100	145 (45.3)
90	109 (34.1)
80	52 (16.3)
70	14 (4.4)
Baseline LDH level, *n* (%)	
≤ 1.5 × ULN	301 (94.1)
> 1.5 × ULN	15 (4.7)
Missing	4 (1.3)
Prior radiotherapy, *n* (%)	
No prior radiotherapy	274 (85.6)
Prior radiotherapy	46 (14.4)
Prior nephrectomy, *n* (%)	
No prior nephrectomy	100 (31.3)
Prior nephrectomy	220 (68.8)
Time from initial diagnosis to randomization, *n* (%)	
< 1 year	208 (65.0)
≥ 1 year	111 (34.7)
Missing	1 (0.3)
Prior adjuvant or neo-adjuvant therapy for localized or locally advanced RCC, *n* (%)	
No prior adjuvant or neo-adjuvant therapy	317 (99.1)
Prior adjuvant or neo-adjuvant therapy	3 (0.9)
Baseline PD-L1 status, *n* (%)	
< 1% or indeterminate	238 (74.4)
≥ 1%	82 (25.6)
Baseline IMDC prognostic score, *n* (%)	
0	74 (23.1)
1–2	185 (57.8)
3–6	61 (19.1)
Liver metastasis, *n* (%)	
No liver metastasis	315 (98.4)
Presence of liver metastasis	5 (1.6)

^a^Sum of the diameter of target lesions at baseline

*IMDC* International Metastatic RCC Database Consortium, *LDH* lactate dehydrogenase, *N* number of patients, *PD-L1* programmed death-ligand 1, *RCC* renal cell carcinoma, *SD* standard deviation, *ULN* upper limit of normal

**Fig. 1:**
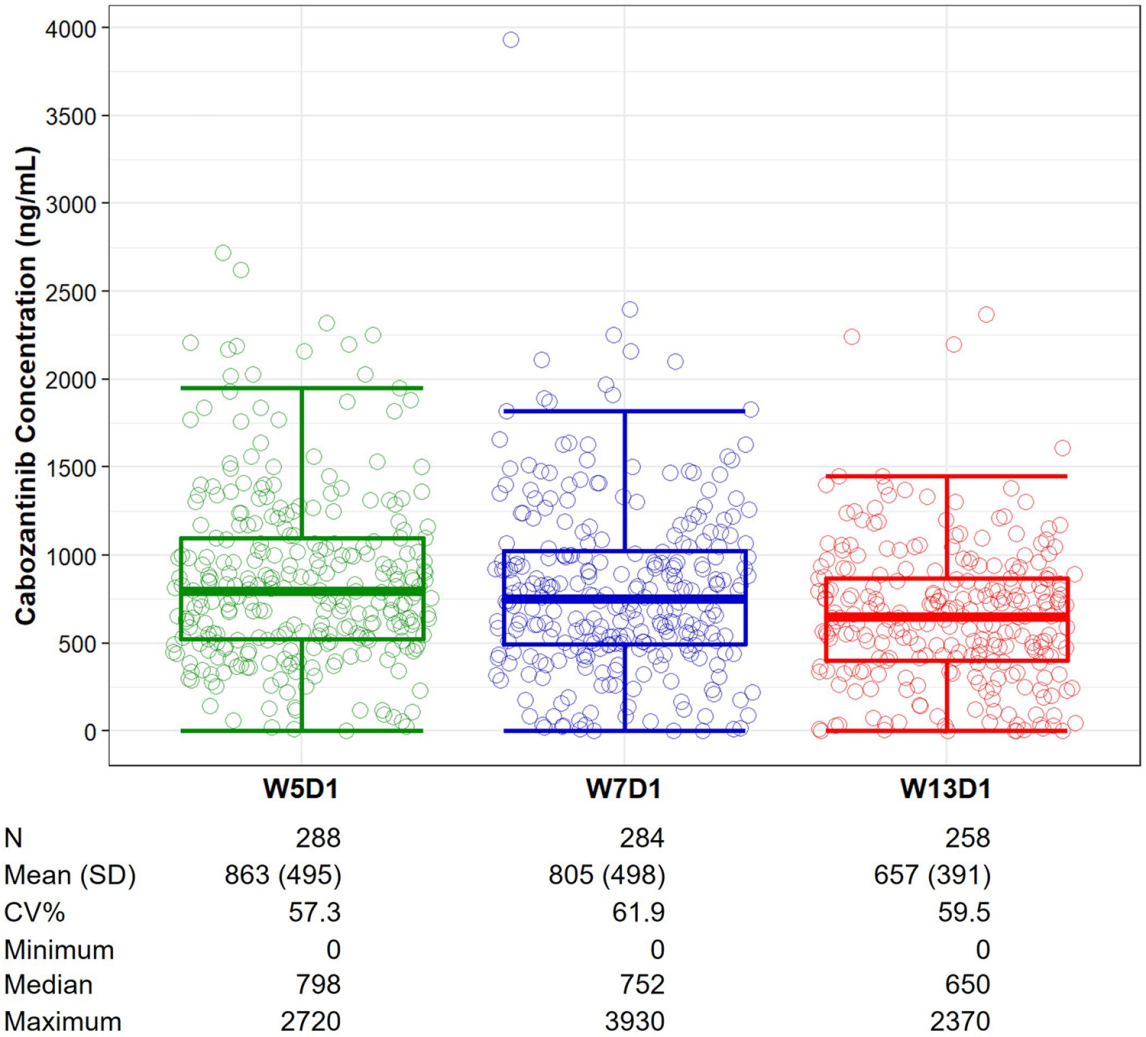
Individual and summary cabozantinib concentrations at scheduled visits. Circles represent the observed cabozantinib concentrations for individual patients. Boxplot display first quartile, median, third quartile, upper error bar (third quartile plus 1.5 times interquartile range), and lower error bar (first quartile minus 1.5 times interquartile range). Circles outside error bar are outliers. *CV%* coefficient of variation, *SD* standard deviation, *W5D1* Week 5 Day 1, *W7D1* Week 7 Day 1; *W13D1* Week 13 Day 1

Analysis of cabozantinib plasma concentrations generally showed decreases over time from a median of 798 ng/mL at Week 5 to 650 ng/mL at Week 13 (Fig. 1). The dose normalized median concentration at Week 5 in the CheckMate 9ER study (19.9 ng/mL/mg) was consistent with that of the METEOR study (20.3 ng/mL/mg), in which patients with RCC received cabozantinib 60 mg/day as monotherapy [17]. This indicates linear cabozantinib PK between 40 mg and 60 mg dose and that nivolumab did not affect cabozantinib PK in RCC patients.

The updated integrated PopPK analysis included 10,333 quantifiable cabozantinib concentrations obtained from 2331 subjects, including 823 cabozantinib concentrations from 308 patients in the CheckMate 9ER trial. A summary of baseline demographics and covariates for the 308 patients from CheckMate 9ER is presented in Supplementary Table S1 (data for the other 10 cabozantinib studies have been previously reported [16]).

PK parameter estimates (Supplementary Table S2) and covariate effects from the updated PopPK model were consistent with the previous PopPK model [16]. The updated population PK analysis demonstrated that patients with RCC who received cabozantinib and nivolumab had cabozantinib exposure comparable to those who received cabozantinib monotherapy. None of the demographic covariates (e.g., age, body weight, sex, and race) appeared to clinically impact cabozantinib exposure. Nivolumab had no significant effect on cabozantinib CL/F. Compared with the reference group (white, healthy male, receiving cabozantinib as a single-agent therapy), the CL/F ratio for the addition of nivolumab was 0.992 (95% CI 0.912–1.08). Results of prediction-corrected VPC demonstrated good predictive performance of the updated PopPK model for CheckMate 9ER (Supplementary Fig. S1).

### Exposure–response analyses

For exposure–response analyses of PFS and safety endpoints, the number of patients in the CheckMate 9ER trial with events and the total number at risk are listed in Supplementary Table S3. These patients received at least one dose of nivolumab plus cabozantinib and had a baseline and/or at least one post-baseline assessment of the clinical endpoint as indicated; patients without a measurable PK concentration were assigned population-level PK parameter estimates. The PK parameters were adjusted for covariates in the 12 patients without measurable PK concentrations for population-level exposures. Ten of these patients had a cabozantinib dose modification event or were censored prior to the PK assessment of cabozantinib at Cycle 3. Although using the population level PK estimates for patients without measurable cabozantinib PK could bias the results given early dose modification or censorship, sensitivity analyses were performed that excluded these 12 patients. Parameter estimates and inferences for the sensitivity analyses did not change the results. The VPC plots using the final CPH model parameters for predicting the incidence of the clinical endpoints are provided in Supplementary Figs. S2–S5.

#### Progression-free survival

**Fig. 2:**
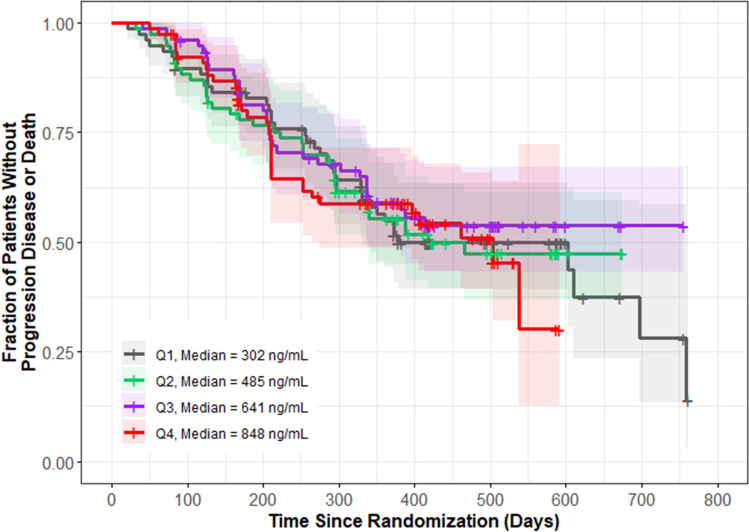
Kaplan–Meier plot for progression-free survival by average exposure quartiles. Shaded regions represent 95% confidence intervals for each exposure quartile (Q#) of CAVG0T, the predicted average cabozantinib concentration from time zero to the event or censoring

Figure 2 presents the exploratory KM plot for PFS by average cabozantinib exposure quartile. The plot includes 311 patients with 144 patients experiencing disease progression or death. There was no clear difference between different quartiles of cabozantinib exposure and the fraction of patients with progressive disease or death.

A linear CPH model including all 311 patients was used to assess the relationship between disease progression or death and cabozantinib exposure (Supplementary Table S4). This model did not produce a statistically significant reduction in − 2LL compared to the model without cabozantinib exposure, indicating no relationship between cabozantinib exposure and PFS. Therefore, a nonlinear model was not tested.

The parameter estimate in the linear PFS base model was used to predict the relative hazard rate for different cabozantinib dose levels. The predicted cabozantinib average concentrations and corresponding dose levels were 386 ng/mL for 20 mg QD, 772 ng/mL for 40 mg QD, and 1157 ng/mL for 60 mg QD. These exposures are based on cabozantinib doses that are constant over the course of the study and do not reflect the observed data where cabozantinib dose modifications may have occurred. There was no statistically significant relationship of PFS with cabozantinib exposure at these three average concentrations (Supplementary Fig. S6). Compared with a reference CAVG0T of 772 ng/mL, the HR (95% CI) was 1.00 (0.78–1.27) for CAVG0T 386 ng/mL and 1.00 (0.79–1.28) for CAVG0T 1157 ng/mL.

##### Covariate effects for PFS

**Fig. 3:**
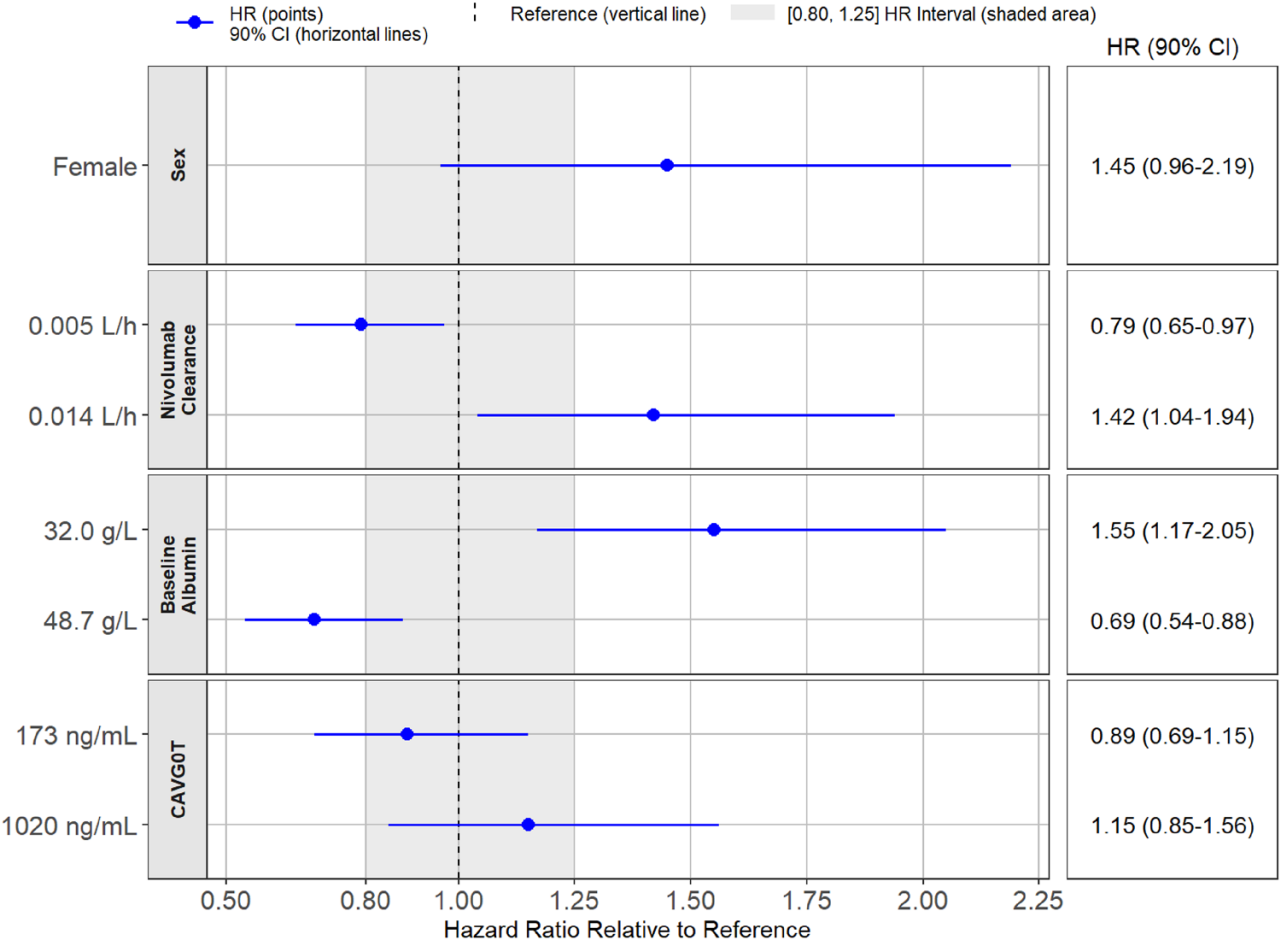
Covariate effects in the final model of progression-free survival model. Reference is a male patient with nivolumab clearance of 0.009 L/h, baseline albumin of 41 g/L, and CAVG0T, the predicted average cabozantinib concentration from time zero to the event or censoring, of 558 ng/mL. Continuous covariates are tested at the 5th and 95th percentile values. Hazard ratio (HR) estimate along with 90% confidence interval (CI) represents the change in log(HR) per unit change in the continuous variable, or the difference in log(HR) between comparator/reference variables (categorical), assuming all other covariates are constant

Table 1 lists the covariates considered for PFS exposure–response analyses. Cabozantinib exposure and the covariates were added simultaneously to the base model and fit using CPH methodology, with the exceptions of prior adjuvant or neo-adjuvant therapy for localized or locally advanced RCC (*n* = 3), and liver metastasis (*n* = 5) due to an insufficient number of patients. Sex, nivolumab clearance, and baseline albumin were included in the final model along with cabozantinib exposure. In the final model, patients with higher baseline nivolumab clearance or lower baseline albumin levels (Pearson correlation, *r* = − 0.4) had a higher predicted rate of disease progression or death (Fig. 3; Supplementary Table S5). There was no significant association of cabozantinib exposure or sex with PFS (*p* > 0.05).

#### Safety endpoints

##### Dose modifications

**Fig. 4:**
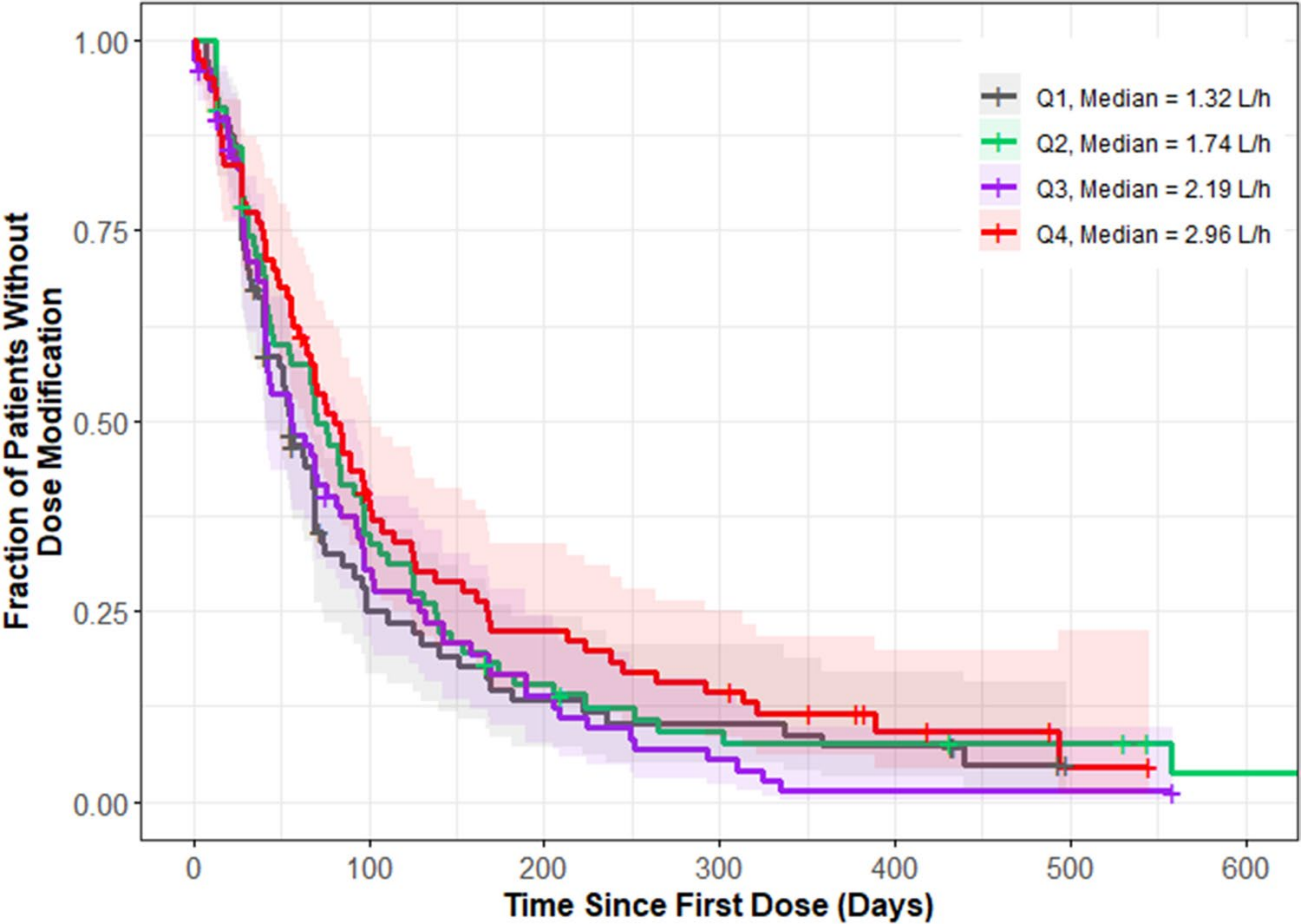
Kaplan–Meier plot for cabozantinib dose modification by individual predicted apparent clearance quartiles. Shaded regions represent 95% confidence intervals for each exposure quartile (Q#) of CL/F, the individual predicted apparent cabozantinib clearance from population pharmacokinetic model

Among patients who received nivolumab plus cabozantinib, 318 were included in the analysis to characterize the relationship between individual predicted cabozantinib CL/F and the rate of dose modifications (Supplementary Table S3); 285 had a dose modification. The exploratory KM plot for cabozantinib dose modification by CL/F quartiles (Fig. 4) shows a slight trend of decreasing frequency of cabozantinib dose modification with higher cabozantinib clearance (lower exposure). However, there was no significant association between dose modification and cabozantinib CL/F based on a CPH model (Supplementary Table S4). The parameter estimate in the selected log-linear base model was used to calculate the relative hazard rate. Relative to a CL/F of 2.2 L/h (approximate typical cabozantinib CL/F value for RCC patients in the PopPK model), the HR (95% CI) was 1.15 (0.93–1.41) for a lower CL/F of 1.2 L/h and 0.92 (0.81–1.04) for a higher CL/F of 3.2 L/h (Supplementary Fig. S7).

##### Adverse events

**Fig. 5:**
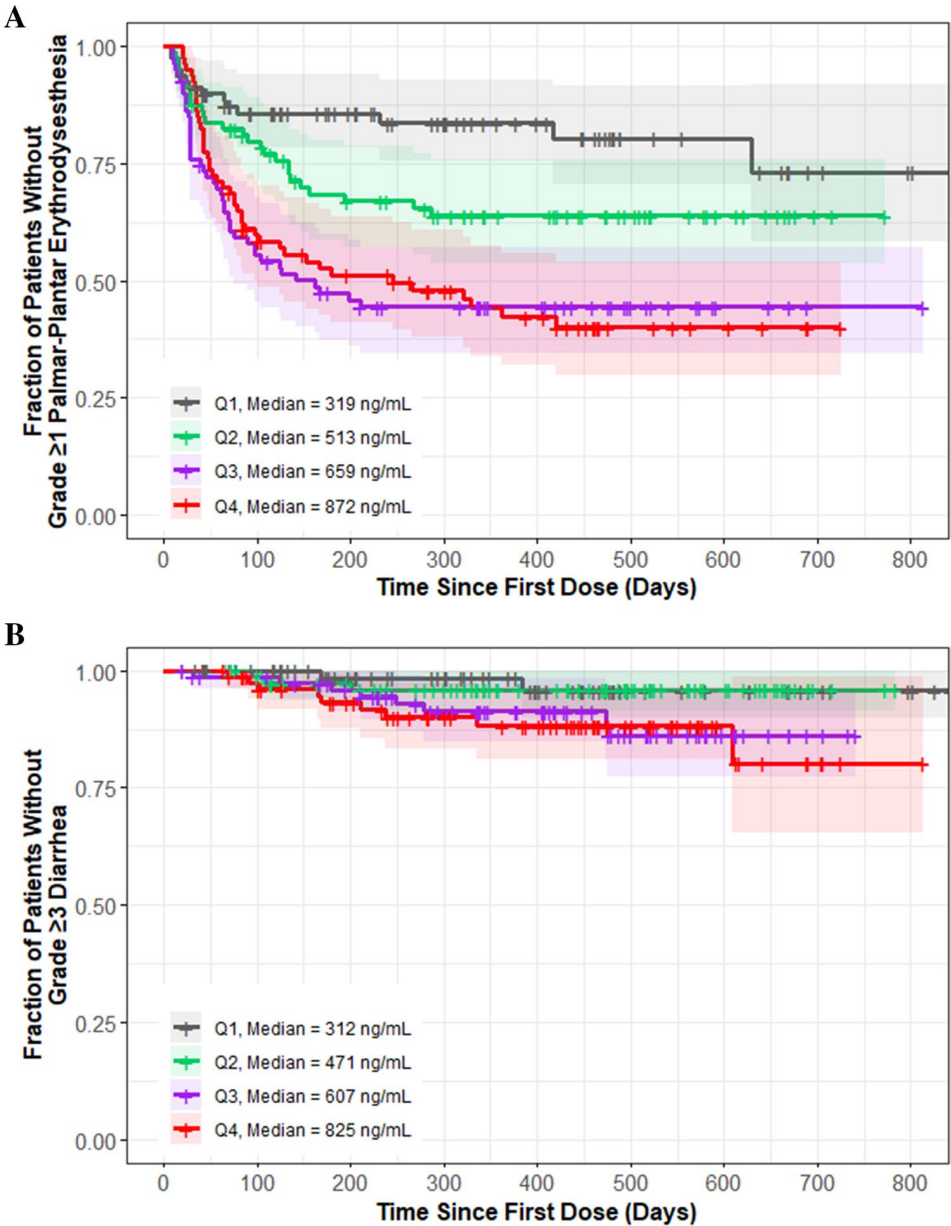
Kaplan–Meier Plot for Grade ≥ 1 palmar-plantar erythrodysesthesia (**A**) and Grade ≥ 3 diarrhea (**B**) by average cabozantinib exposure quartiles. Shaded regions represent 95% confidence intervals for each exposure quartile (Q#) of CAVG0T, the predicted average cabozantinib concentration from time zero to the event or censoring

Among the AE endpoints evaluated, exploratory KM plots by exposure quartile indicated an association of cabozantinib exposure with PPE (Grade ≥ 1) and diarrhea (Grade ≥ 3) but not with the other AE endpoints. The exploratory KM plots for PPE (Fig. 5a) and for diarrhea (Fig. 5b) showed that the fraction of patients who experienced either of these AEs generally increased with higher cabozantinib exposure quartiles. Both linear and nonlinear models for PPE and diarrhea demonstrated a statistically significant relationship with cabozantinib exposure (Supplementary Table S4), although the estimated EC_50_ for both endpoints in the nonlinear base model was outside the average concentration range at 20 mg or 40 mg daily dose. Given that the EC_50_ values in the nonlinear form were out of range, the significant linear model was selected to describe the rate of PPE and diarrhea with cabozantinib exposure. Relative to a reference cabozantinib CAVG0T of 772 ng/mL at 40 mg dose in the linear base model, HRs (95% CI) for CAVG0T 386 ng/mL at 20 mg dose were 0.63 (0.50–0.78) for PPE and 0.48 (0.29–0.80) for diarrhea (Supplementary Figs. S8 and S9). Statistically significant exposure–response relationships were not found for hypertension (Grade ≥ 3), fatigue/asthenia (Grade ≥ 3), or rate of ALT/AST elevation (Grade ≥ 3) in the linear base model (Supplementary Table S4).

## Discussion

The CheckMate 9ER trial investigated the efficacy and safety of nivolumab in combination with cabozantinib compared with sunitinib in patients with previously untreated advanced RCC [11]. Patients received nivolumab 240 mg intravenously every 2 weeks plus cabozantinib 40 mg orally QD. Patients were allowed to hold and/or reduce cabozantinib dosing to manage AEs, and dose holds were allowed for nivolumab. The cabozantinib dose could be reduced from 40 to 20 mg QD and then to 20 mg QOD.

Nivolumab plus cabozantinib significantly improved the primary efficacy endpoint of PFS compared with sunitinib [11]. In contrast to the METEOR study, the population exposure–response analysis in patients with RCC in the CheckMate 9ER study did not find a relationship between PFS and average cabozantinib concentration. This difference is likely due to the CPH methodology with time-varying measures used in the METEOR study [17]. For the METEOR study, the ER relationship using the CPH model was not demonstrated through descriptive analysis (data on file), but rather through the simulation approach. The CPH modeling with time-varying measures used for METEOR potentially overestimated the significant effect of the exposure matrix on PFS. To avoid this bias, the average cabozantinib concentration from time 0 to the first event (CAVG0T) was used for ER analysis of PFS in CheckMate 9ER. Individual post hoc predicted CL/F values from the PopPK analysis were used as the exposure metric for evaluating dose modification, as it can be treated as a baseline exposure characteristic if a patient were to receive uninterrupted daily cabozantinib.

In patients receiving the combination, 68.1% had at least one cabozantinib dose hold and 56.3% had a reduction in their cabozantinib dose, but the rate of discontinuations due to adverse events was low (7.5% of patients discontinued cabozantinib only due to AEs), indicating effective AE management with dose modification to maintain tolerability [11]. In the CheckMate 9ER trial, the median time to first dose hold (68 days), and the median time to first-level dose reduction (106 days) [18], occurred much later than 38 days for first dose hold and 55 days for first-level dose reduction in the METEOR study [17], where cabozantinib 60 mg QD was assessed in patients with metastatic RCC as single-agent therapy. In the exposure–response analysis, the relationship between individual predicted cabozantinib CL/F and the rate of cabozantinib dose modifications assessed with CPH models was not statistically significant in the CheckMate 9ER study with cabozantinib 40 mg QD but was statistically significant in the METEOR study with cabozantinib 60 mg QD [14]. The non-significant association between cabozantinib CL/F and the rate of dose modification in CheckMate 9ER indicates that cabozantinib 40 mg QD allows patients to reach optimal concentrations in combination with nivolumab and maintain tolerability.

Exploratory KM plots showed no clear difference between quartiles of cabozantinib exposure and the fraction of patients receiving the combination in CheckMate 9ER with progressive disease or death, but higher cabozantinib exposure was associated with increased risk of PPE (Grade ≥ 1) and diarrhea (Grade ≥ 3). Simulation using the final CPH model parameters estimates for a 20-mg, 40-mg, or 60-mg dose of cabozantinib also showed no statistically significant relationship with disease progression or death, while a dose of 20 mg vs. 40 mg was predicted to reduce the risk of PPE and diarrhea. Taken together, these data support the dosing strategy of cabozantinib when used in combination with nivolumab—initiate the cabozantinib dose at 40 mg/day to ensure efficacious cabozantinib exposure across the spectrum of cabozantinib clearance in a patient population: patients with high cabozantinib clearance are predicted to maintain tolerability at the efficacious exposure level without the need for dose modification; patients with low cabozantinib clearance are predicted to require dose modification to manage tolerability, and dose reduction to 20 mg/day will maintain cabozantinib exposure at an efficacious level. These findings are consistent with previous exposure–response analyses of single-agent cabozantinib [9]. Although dose reduction to 20 mg QOD was allowed to manage AEs in CheckMate 9ER, only 8% of patients received this dose, resulting in insufficient data for a reliable simulation in an exposure–response analysis [19].

ALT/AST increases, and diarrhea, hypothyroidism, and rash are part of known safety profiles of both cabozantinib and nivolumab and were more frequently reported with the combination than with sunitinib in CheckMate 9ER study [11], suggesting potential overlapping toxicity with the combination. In the current analysis, cabozantinib exposure was a significant predictor for diarrhea but not ALT/AST elevation (Grade ≥ 3). The incidence of Grade ≥ 3 hypothyroidism and rash were low (< 2%), and therefore, these were not included in the exposure–response analyses.

An increased risk of hypertension (systolic BP > 160 mmHg or diastolic BP > 100 mmHg) and fatigue/asthenia at higher cabozantinib concentrations were reported with single-agent cabozantinib 60 mg QD in the METEOR study [14]. These relationships were not observed with cabozantinib 40 mg QD in combination with nivolumab in CheckMate 9ER. The lower rate of hypertension (systolic BP > 160 mmHg or diastolic BP > 100 mmHg) in the combination arm of CheckMate 9ER compared with the cabozantinib arm of METEOR (14% vs. 32%) and the lack of associations between cabozantinib exposure and other safety endpoints in CheckMate 9ER further support a cabozantinib dose of 40 mg QD in combination with nivolumab based on tolerability.

Covariate analysis for CPH modeling of PFS indicated a higher predicted rate of disease progression or death with lower baseline albumin level or higher baseline nivolumab clearance but not with other baseline covariates for patients with advanced RCC. These findings are consistent with results from previous analysis of nivolumab. In a prospective cohort study of patients receiving nivolumab monotherapy for non-small cell lung cancer (NSCLC), melanoma, or RCC, exposure–response analyses demonstrated a significant clearance–response relationship in NSCLC, a non-significant trend in RCC, and no relationship in melanoma [20]. Of note, this study also predicted that low albumin at baseline and higher nivolumab clearance are significant covariates associated with worse PFS. Hypoalbuminemia is a well-known marker of cachexia, inflammatory conditions, and increased catabolic activity. Increased catabolic activity is associated with degradation of IgG and, consequently, increased clearance and reduced systemic exposure of therapeutic monoclonal antibodies (mAbs) such as nivolumab [21]. Since catabolic degradation of mAbs is a function of systemic inflammatory status, it may change over time as a function of response to therapy [21]. Time-varying clearance has been demonstrated for nivolumab and other therapeutic antibodies [21, 22]. In an analysis where change of clearance over time was associated with post-treatment effects, nivolumab clearance decreased when disease status improved [22].

In summary, the exposure–response analysis reported here further supports the dosing strategy implemented in CheckMate 9ER: administering cabozantinib at 40 mg orally QD in combination with a standard dose of nivolumab to ensure all patients receive an efficacious dose of cabozantinib regardless of cabozantinib clearance variability (i.e., both high and low clearance) and using dose modification to manage AEs. Reducing cabozantinib exposure was not predicted to significantly impact the PFS benefit in patients with advanced RCC treated with nivolumab plus cabozantinib in the CheckMate 9ER trial, but was predicted to reduce the risk of PPE and diarrhea, two of the more common AEs associated with cabozantinib. Thus, the dose of cabozantinib can be reduced from 40 mg QD to 20 mg QD in patients requiring a dose reduction (i.e., patients with low cabozantinib clearance) to improve tolerability without adversely affecting efficacy. Further, administering cabozantinib at 60 mg QD instead of 40 mg QD in combination with nivolumab was not predicted to improve PFS.

## Supplementary Information

Below is the link to the electronic supplementary material.

Supplementary file1 (DOCX 503 KB)

## Data Availability

Inquiries can be directed to the corresponding author.

## Code availability

Inquiries can be directed to the corresponding author.
