# Supplementary material for: Cabozantinib exposure–response analysis for the phase 3 CheckMate 9ER trial of nivolumab plus cabozantinib versus sunitinib in first-line advanced renal cell carcinoma
Source: Cancer Chemother Pharmacol. 2023 Jan 10;91(2):179–89. doi: 10.1007/s00280-022-04500-9 (PMC9905187; doi:10.1007/s00280-022-04500-9)
Supplement: Supplementary file 1 — Supplementary file1 (DOCX 503 KB) [file 280_2022_4500_MOESM1_ESM.docx]

**SUPPLEMENTAL MATERIALS**

**Cabozantinib Exposure-Response Analysis for the Phase 3 CheckMate 9ER Trial of Nivolumab Plus Cabozantinib Versus Sunitinib in First-Line Advanced Renal Cell Carcinoma**

Benjamin Duy Tran, Jing Li, Neang Ly, Raffaella Faggioni, Lorin Roskos

**Corresponding author:** Benjamin Duy Tran, PharmD

Email: [btran@exelixis.com](mailto:btran@exelixis.com)

**Table of Contents**

[Table S1 Baseline demographics and covariates for CheckMate 9ER used in the updated integrated population pharmacokinetic model of cabozantinib (patients with at least one measurable cabozantinib concentration) 2](#_Toc121315116)

[Table S2 Parameter estimates (95% CI) for the updated cabozantinib integrated pharmacokinetic model 3](#_Toc121315117)

[Table S3 Summary of number of events and number of patients at risk for time-to-event endpoints for study CheckMate 9ER 5](#_Toc121315118)

[Table S4 Cox proportional hazard models and parameters for exposure-response analyses 6](#_Toc121315119)

[Table S5 Parameter estimates for final progression-free survival model in exposure-response analyses 7](#_Toc121315120)

[Fig. S1 Prediction-corrected visual predictive check for CheckMate 9ER 8](#_Toc121315121)

[Fig. S2 Visual predictive check for the model of progression-free survival 9](#_Toc121315122)

[Fig. S3 Visual predictive check for the model of cabozantinib dose modification 10](#_Toc121315123)

[Fig. S4 Visual predictive check for the model of palmar-plantar erythrodysesthesia 11](#_Toc121315124)

[Fig. S5 Visual predictive check for the model of diarrhea 12](#_Toc121315125)

[Fig. S6 Predicted survival curves for progression-free survival at constant average cabozantinib concentrations based on 20 mg, 40 mg, and 60 mg doses 13](#_Toc121315126)

[Fig. S7 Predicted fractions of patients without cabozantinib dose modification for selected values of cabozantinib apparent clearance (CL/F) 14](#_Toc121315127)

[Fig. S8 Predicted fractions of patients without palmar-plantar erythrodysesthesia (Grade ≥1) at specific, constant average cabozantinib concentrations 15](#_Toc121315128)

[Fig. S9 Predicted fractions of patients without diarrhea (Grade ≥3) at specific, constant average cabozantinib concentrations 16](#_Toc121315129)

# Table S1 Baseline demographics and covariates for CheckMate 9ER used in the updated integrated population pharmacokinetic model of cabozantinib (patients with at least one measurable cabozantinib concentration)

|  | **CheckMate 9ER**  ***N* = 308** |
| --- | --- |
| **Sex, n (%)** |  |
| Male | 237 (76.9) |
| Female | 71 (23.1) |
| **Race, n (%)** |  |
| White | 253 (82.1) |
| Black | 1 (0.3) |
| Asian | 26 (8.4) |
| Other | 28 (9.1) |
| **Body Weight (kg)** |  |
| N | 308 |
| Mean (SD) | 81.6 (17.8) |
| Median (range) | 80.2 (36.0−160.4) |
| **Age (years)** |  |
| Mean (SD) | 61.3 (10.3) |
| Median (range) | 62 (29–90) |
| **ALT (U/L)** |  |
| N | 307 |
| Mean (SD) | 22.6 (14.9) |
| Median (range) | 18 (5–102) |
| **AST (U/L)** |  |
| N | 307 |
| Mean (SD) | 21.1 (9.8) |
| Median (range) | 19 (6–69) |
| **Total Bilirubin (µmol/L)** |  |
| N | 307 |
| Mean (SD) | 8.93 (4.3) |
| Median (range) | 8.6 (2.6–39.3) |
| **Creatinine Clearance (mL/min)^a^** |  |
| N | 307 |
| Mean (SD) | 77.7 (27.8) |
| Median (range) | 72.2 (27.2–196.5) |
| ^a^Creatinine clearance was estimated using the Cockcroft-Gault equation  *ALT* alanine aminotransferase, *AST* aspartate aminotransferase, *N* number, *SD* standard deviation | |

# Table S2 Parameter estimates (95% CI) for the updated cabozantinib integrated pharmacokinetic model

| Parameters | Transformed Estimate | 95% CI |
| --- | --- | --- |
| PK parameters | | |
| Ka (hr^-1^) | 1.13 | (0.683–1.88) |
| Duration of zero-order absorption (hr) | 2.42 | (2.02–2.89) |
| CL/F (L/hr) | 2.35 | (2.14–2.59) |
| Vc/F (L) | 182 | (154–216) |
| Q/F (L/hr) | 30.3 | (27.3–33.7) |
| Vp/F (L) | 177 | (165–190) |
| ALAG1 (hr) | 0.815 | (0.786–0.845) |
| Fraction of dose in first absorption depot F1^a^ | 0.846 | (0.801–0.883) |
| Dose dependent Ka | 0.496 | (-0.0465–1.04) |
| Covariates | | |
| Capsule on Ka^b^ | 0.628 | (0.241–1.64) |
| Capsule on overall relative oral availability^b^ | 0.834 | (0.813–0.855) |
| Age on CL/F | -0.197 | (-0.312–-0.0826) |
| Female on CL/F^b^ | 0.765 | (0.717–0.817) |
| Black on CL/F^b^ | 1.16 | (1.01–1.34) |
| Asian on CL/F^b^ | 0.899 | (0.830–0.974) |
| Other Race on CL/F^b^ | 0.973 | (0.861–1.10) |
| Weight on CL/F | -0.0443 | (-0.159–0.0706) |
| RCC^c^ on CL/F^b^ | 0.927 | (0.827–1.04) |
| CRPC on CL/F^b^ | 1.04 | (0.935–1.17) |
| MTC on CL/F^b^ | 1.98 | (1.76–2.22) |
| GB on CL/F^b^ | 1.25 | (1.01–1.54) |
| Other malignancies on CL/F^b^ | 1.22 | (1.01–1.48) |
| Age on Vc/F | -0.0692 | (-0.300–0.162) |
| Female on Vc/F^b^ | 1.11 | (0.959–1.28) |
| Black on Vc/F^b^ | 1.07 | (0.743–1.56) |
| Asian on Vc/F^b^ | 0.612 | (0.469–0.799) |
| Other Race on Vc/F^b^ | 0.786 | (0.522–1.18) |
| Weight on Vc/F^b^ | 1.17 | (0.861–1.48) |
| RCC^d^ on Vc/F^b^ | 0.438 | (0.156–1.23) |
| CRPC on Vc/F^b^ | 0.860 | (0.690–1.07) |
| MTC on Vc/F^b^ | 1.03 | (0.858–1.25) |
| GB on Vc/F^b^ | 0.457 | (0.278–0.750) |
| Other malignancies on Vc/F^b^ | 0.884 | (0.668–1.17) |
| HCC on CL/F^b^ | 0.938 | (0.840–1.05) |
| HCC on Vc/F^b^ | 1.01 | (0.818–1.23) |
| Nivolumab on CL/F^b^ | 0.992 | (0.912–1.08) |
| Variance | | |
| σ^2^ | 0.131 | (0.127–0.135) |
| ω^2^ Ka | 2.52 | (1.21–3.83) |
| ω^2^ CL/F | 0.201 | (0.186–0.217) |
| ω^2^ CL/F:Vc/F | 0.187 | (0.150–0.224) |
| ω^2^ Vc/F | 0.432 | (0.350–0.513) |
| ω^2^ F1 | 2.69 | (1.97–3.42) |

^a^Anti-logit transformation was used to obtain F1

^b^For categorical covariates (e.g., capsule), transformed estimates correspond to fractional change from the reference level

^c^RCC for CL/F includes patients in Studies XL184-308 (METEOR) and CheckMate-9ER

^d^RCC for Vc/F includes only patients from Study XL184-308 (METEOR)

Transformed Estimate PK parameter obtained by exponentiating the original estimate, if applicable.

*ALAG1* absorption lag time for the 1^st^ absorption depot, *CI* confidence interval, *CL/F* apparent clearance, *CRPC* castration-resistant prostate cancer, *GB* glioblastoma multiforme, *HCC* hepatocellular carcinoma, *Ka* absorption rate constant from the 1^st^ absorption depot, *MTC* metastatic medullary thyroid cancer, *Q/F* apparent flow parameter between compartments, *RCC* renal cell carcinoma, *SE* standard error, *Vc/F* apparent distribution volume of the central compartment, *Vp/F* apparent distribution volume of the peripheral compartment, *σ^2^* variance of population predicted concentration, *ω^2^* variance of population parameter

# Table S3 Summary of number of events and number of patients at risk for time-to-event endpoints for study CheckMate 9ER

| **Endpoint** | **Number of Events^a^ (Number of Patients without PK)** | **Total Number of Patients at Risk (Number of Patients without PK)** |
| --- | --- | --- |
| Progression-free survival (progressive disease or death) | 144 (5) | 311^g^ (8) |
| Cabozantinib dose modification^b^ | 285 (6) | 318^h^ (12) |
| PPE^c^ | 128 (0) | 320 (12) |
| Diarrhea^d^ | 22 (1) | 320 (12) |
| Hypertension^e^ | 44 (0) | 320 (12) |
| Fatigue/asthenia^d^ | 24 (1) | 320 (12) |
| ALT/AST elevation^f^ | 35 (1) | 317^i^ (10) |
| ^a^One event counted per endpoint per patient (data cut-off 30 March 2020)  ^b^Dose reductions or holds  ^c^Grade ≥1  ^d^Grade ≥3  ^e^Grade ≥3 (systolic BP >160 mmHg or diastolic BP >100 mmHg)  ^f^Grade ≥ 3 (per CTCAE v4.03 criteria for ALT/AST elevation)  ^g^For the analysis of progression free survival, patients were required to have a valid baseline tumor assessment and at least one evaluable post-baseline tumor assessment; 9 patients did not meet these criteria  ^h^2 patients started at 20 mg (instead of 40 mg per protocol) and were omitted from the cabozantinib dose modification analysis  ^i^3 patients did not have valid ALT/AST measurements post-baseline  *ALT* alanine aminotransferase, *AST* aspartate aminotransferase, *PK* pharmacokinetics, *PPE* palmar-plantar erythrodysesthesia | | |

# Table S4 Cox proportional hazard models and parameters for exposure-response analyses

| **Endpoint** | **Model** | **Exposure Metric** | **-2LL** | **EC_50_** | **Parameter** | **Estimate** | **Standard Error** | ***p*-value** |  |
| --- | --- | --- | --- | --- | --- | --- | --- | --- | --- |
| Progression-free survival | Linear | CAVG0T | 1365 | ─ | β_CAVG0T_ | 1.21x10^-5^ | 3.2x10^-4^ | 0.97 |  |
| Dose modification | Log-linear  Linear | CL/F  CL/F | 2339  2340 | ─  ─ | β_logCLF_  β_CLF_ | -0.227  -0.0323 | 0.172  0.0679 | 0.19  0.63 |  |
| PPE | Linear  Non-linear | CAVG0T  CAVG0T | 1282  1270 | ─  6 | β_CAVG0T_  β_EXNL_ | 1.21x10^-3^  106 | 2.93x10^-4^  23.6 | <0.01  <0.01 |  |
| Diarrhea | Linear  Non-linear | CAVG0T  CAVG0T | 217  216 | ─  1077 | β_CAVG0T_  β_EXNL_ | 1.9x10^-3^  6.56 | 6.8x10^-4^  2.48 | <0.01  <0.01 |  |
| Hypertension | Linear | CAVG0T | 451 | - | β_CAVG0T_ | -1.27x10^-3^ | 7.53x10^-4^ | 0.09 |  |
| Fatigue/Asthenia | Linear | CAVG0T | 253 | - | β_CAVG0T_ | 5.2x10^-4^ | 8.28x10^-4^ | 0.53 |  |
| ALT/AST elevation | Linear | CAVG0T | 369 | - | β_CAVG0T_ | 9.63x10^-4^ | 6.08x10^-4^ | 0.11 |  |
| *-2LL* negative 2 log likelihood, *ALT/AST* alanine transaminase/aspartate transaminase, *CAVG0T* predicted average cabozantinib concentration from time zero to the event or censoring time (ng/mL), *β_CAVG0T_* the change in the log hazard ratio per unit change in CAVG0T, *βCLF* the change in the hazard ratio per unit change in CL/F for linear model, *EC_50_* drug exposure producing one-half the maximum drug effect, *βEXNL* the maximum change in the log hazard ratio, *EXNL* CAVG0T/(EC50+CAVG0T), *βlogCLF* the change in the log hazard ratio per unit change in log(CL/F) for log-linear model, *PPE* palmar-plantar erythrodysesthesia | | | | | | | | | |

# Table S5 Parameter estimates for final progression-free survival model in exposure-response analyses

| **Parameter** | **Estimate** | **Standard Error** | ***p*-value** |
| --- | --- | --- | --- |
| CAVG0T | 0.000294 | 0.000337 | 0.38 |
| Female sex | 0.371 | 0.210 | 0.08 |
| Baseline albumin level (g/dl) | -0.0480 | 0.0160 | <0.01 |
| Baseline nivolumab clearance (L/hr) | 66.2 | 29.6 | 0.03 |

*CAVG0T* predicted average cabozantinib concentration from time 0 to *t* (ng/mL)

# Fig. S1 Prediction-corrected visual predictive check for CheckMate 9ER


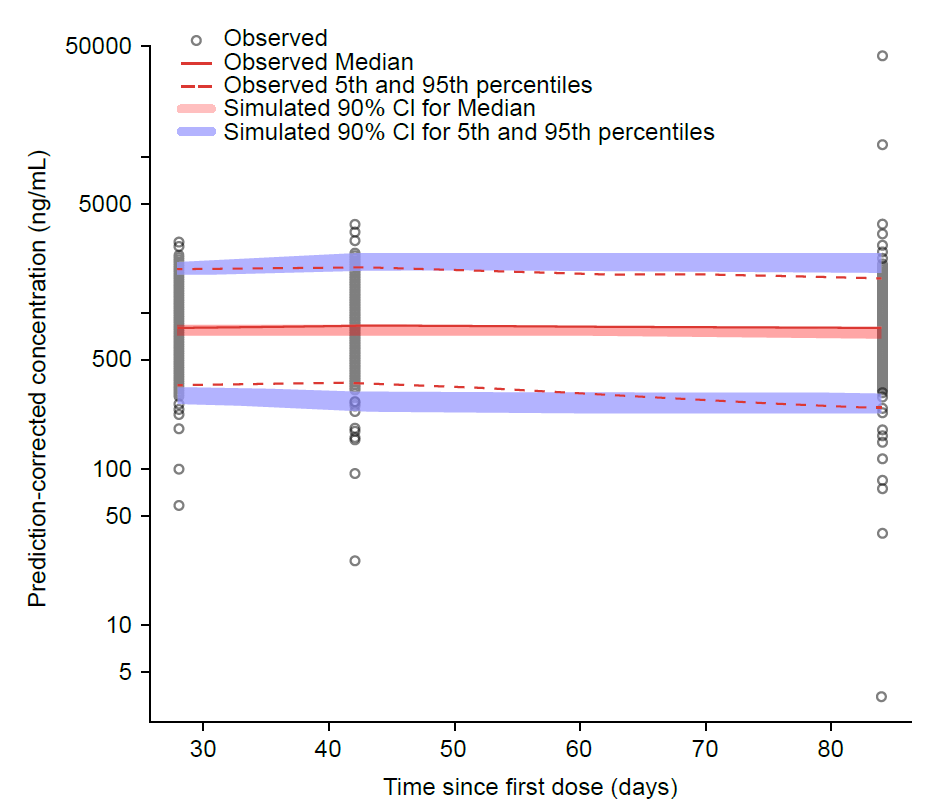


Lower, middle, and upper shaded areas correspond to 90% confidence intervals for the simulated 5th, 50th, and 95^th^ percentiles, respectively. Red solid and dashed lines represent observed data

*CI* confidence interval

# Fig. S2 Visual predictive check for the model of progression-free survival


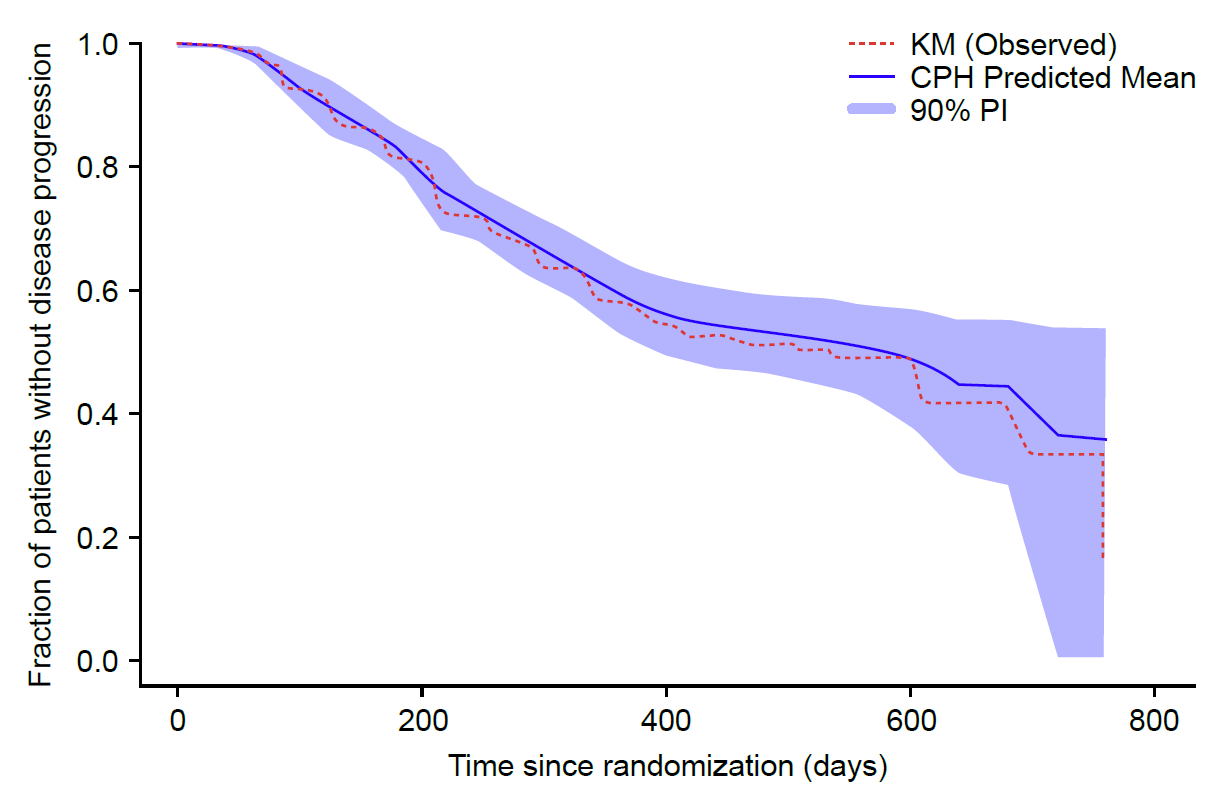


*KM* Kaplan-Meier, *CPH* Cox proportional hazard, *PI* prediction interval

# Fig. S3 Visual predictive check for the model of cabozantinib dose modification


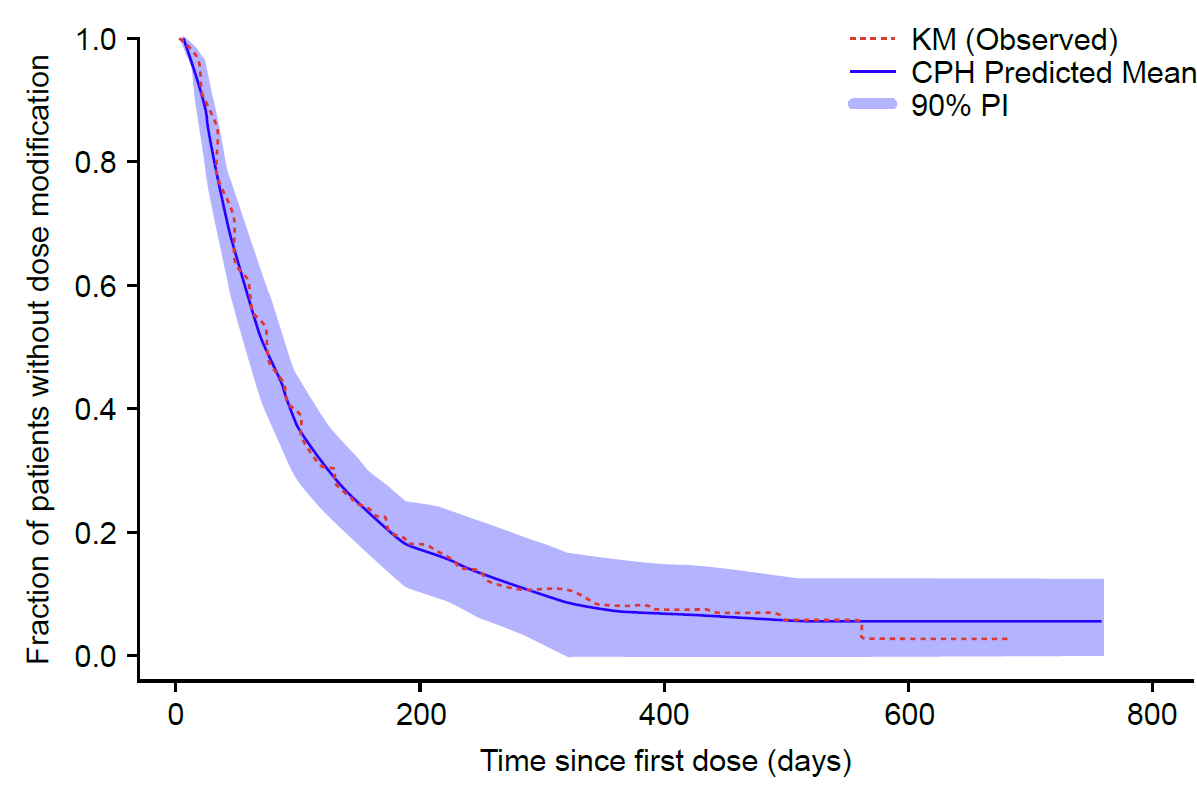


*KM* Kaplan-Meier, *CPH* Cox proportional hazard, *PI* prediction interval

# Fig. S4 Visual predictive check for the model of palmar-plantar erythrodysesthesia


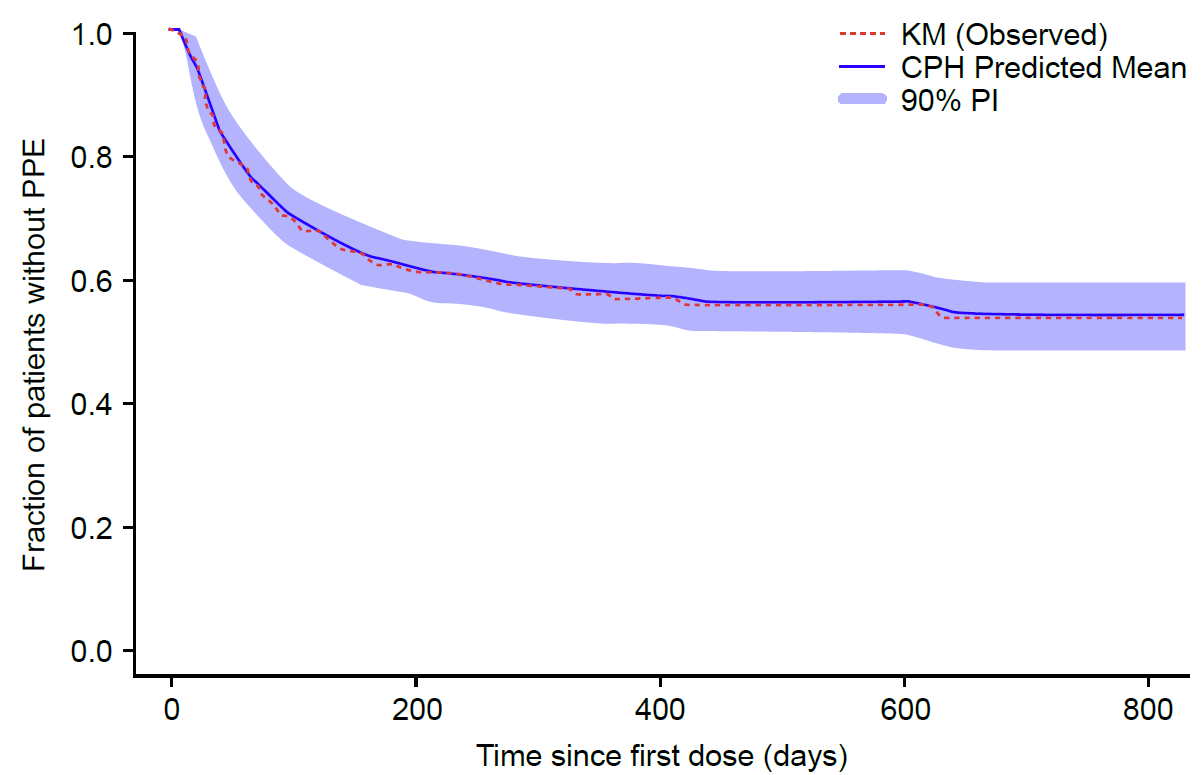


*KM* Kaplan-Meier, *CPH* Cox proportional hazard, *PI* prediction interval

# Fig. S5 Visual predictive check for the model of diarrhea


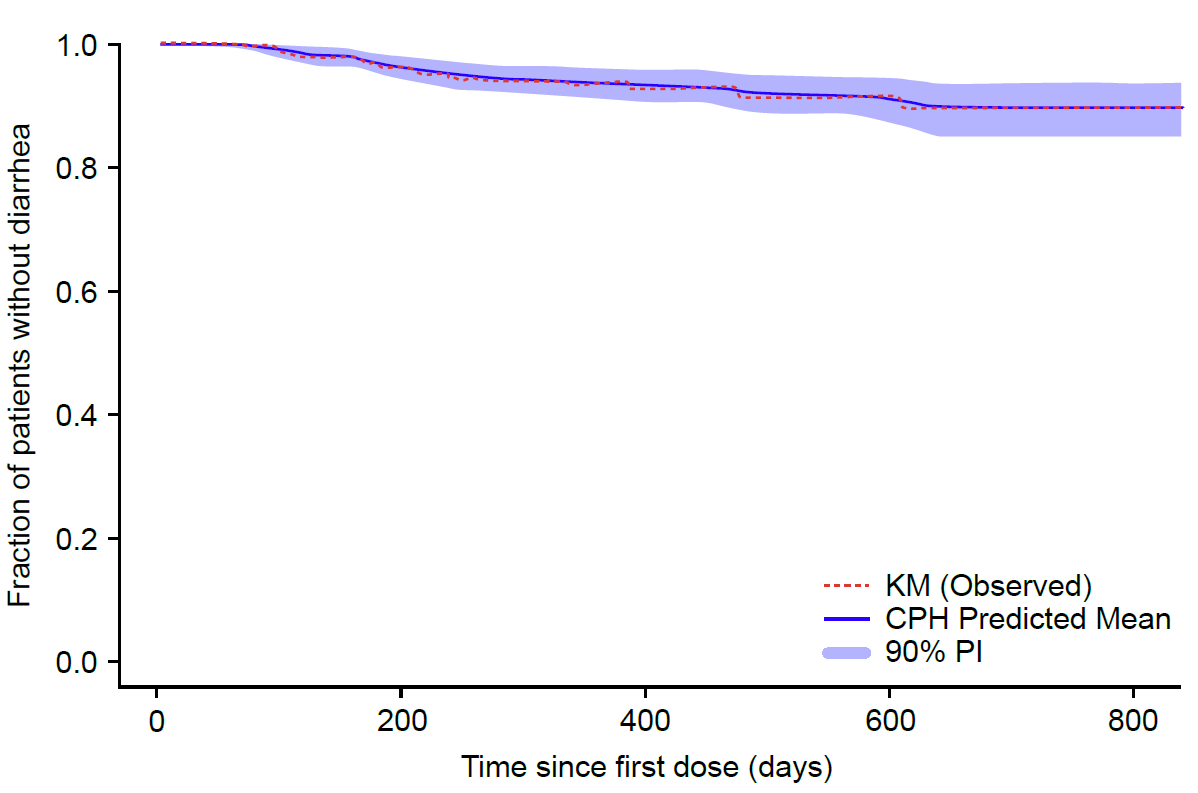


*KM* Kaplan-Meier, *CPH* Cox proportional hazard, *PI* prediction interval

Fig. S6 Predicted survival curves for progression-free survival at constant average cabozantinib concentrations based on 20 mg, 40 mg, and 60 mg doses


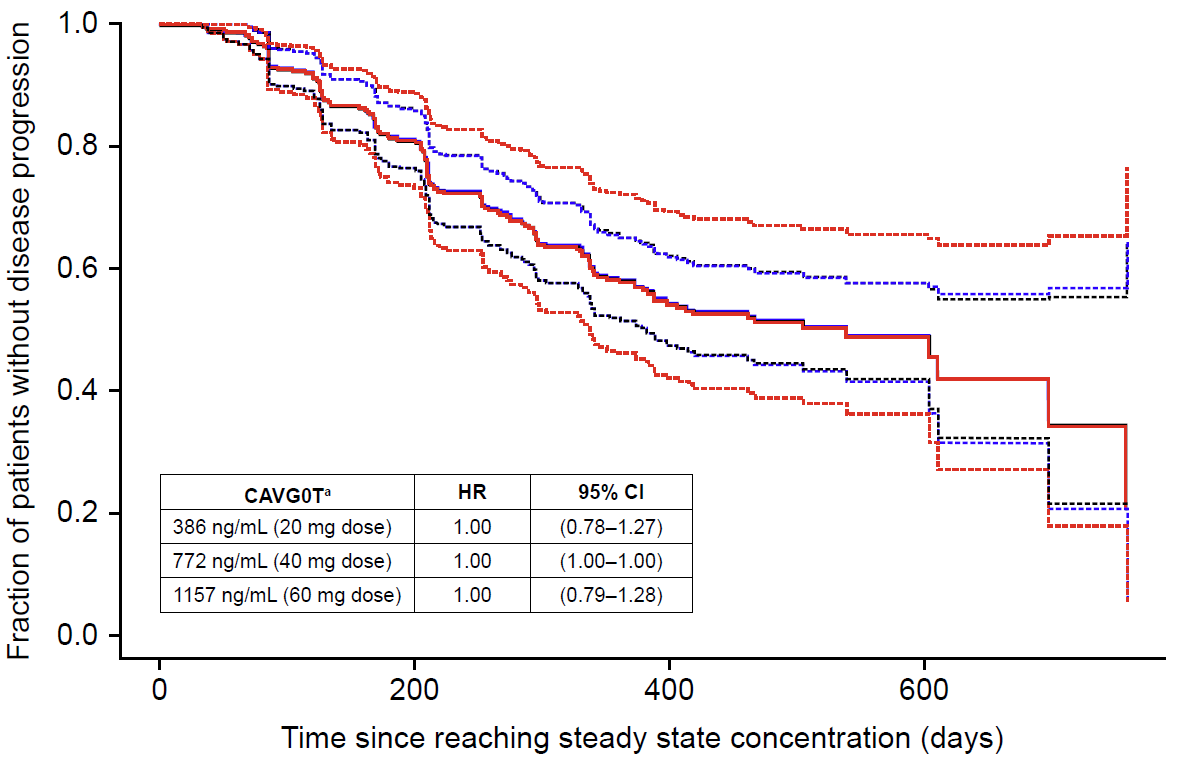


Typical individual predicted steady-state average cabozantinib concentration for the 20 mg (black), 40 mg (blue), and 60 mg (red) doses are 386, 772, and 1157 ng/mL, respectively. The solid line represents the fraction of patients at each dose level without disease progression or death over time. The dashed lines represent 95% confidence intervals

^a^Cabozantinib concentrations correspond to model predicted typical individual steady-state average concentrations for the 20 mg, 40 mg, and 60 mg once daily dosing regimens

*CAVG0T* average cabozantinib concentration calculated from Time 0 to t (ng/mL), *CI* confidence interval, *HR* hazard ratio

# **Fig. S7** Predicted fractions of patients without cabozantinib dose modification for selected values of cabozantinib apparent clearance (CL/F)


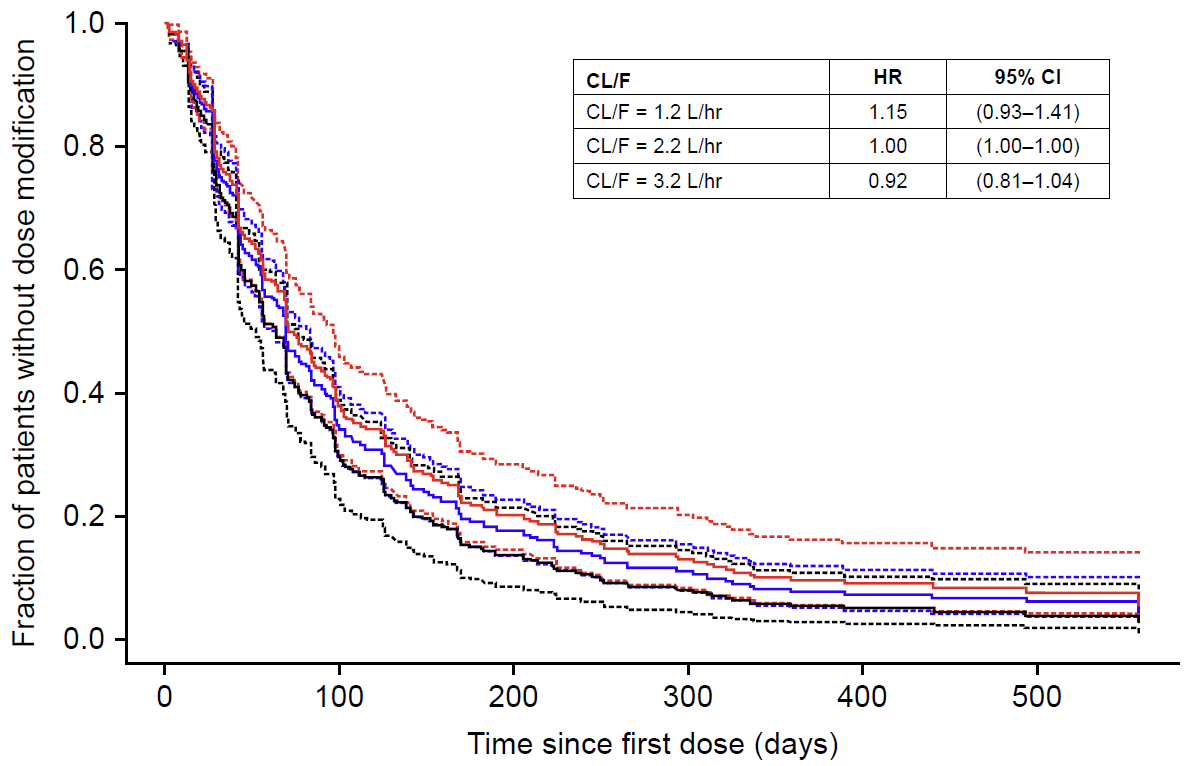


The solid black line (dashed black lines represent 95% CI) represents the fraction of patients without cabozantinib dose modification over time for CL/F of 1.2 L/hr, the solid blue line (dashed blue lines represent 95% CI) represents the fraction of patients without cabozantinib dose modification over time for CL/F of 2.2 L/hr (reference CL/F), and the solid red line (dashed red lines represent 95% CI) represents the fraction of patients without cabozantinib dose modification over time for CL/F of 3.2 L/hr

*CI* confidence interval, *HR* hazard ratio

# **Fig. S8** Predicted fractions of patients without palmar-plantar erythrodysesthesia (Grade ≥1) at specific, constant average cabozantinib concentrations


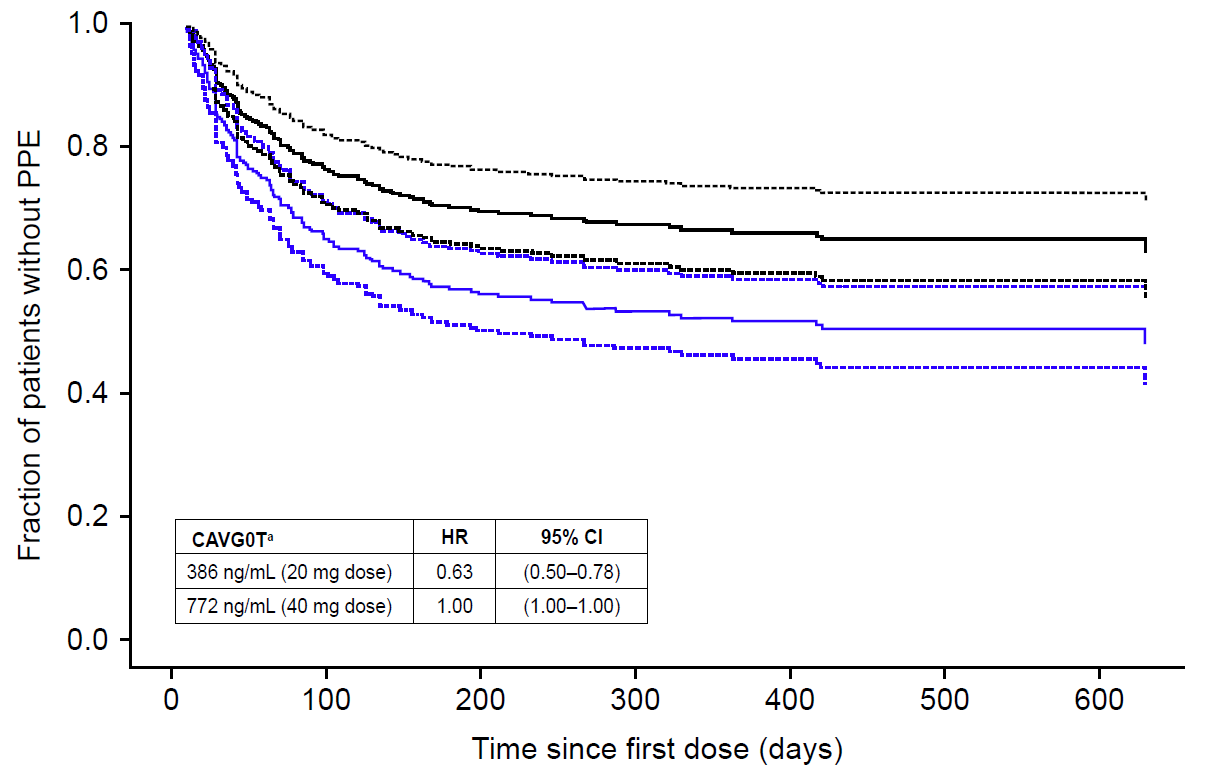


Typical individual predicted steady-state average cabozantinib concentration for the 20 mg (black) and 40 mg (blue, reference CAVG0T) doses are 386 and 772 ng/mL, respectively. The solid line represents the fraction of patients at each dose level without PPE over time. The dashed lines represent 95% confidence intervals

^a^Cabozantinib concentrations correspond to model predicted typical individual steady-state average concentrations for the 20 mg and 40 mg once daily dosing regimens

*CAVG0T* predicted average cabozantinib concentration from time zero to the event or censoring time, *CI* confidence interval, *HR* hazard ratio, *PPE* palmar-plantar erythrodysesthesia

# **Fig. S9** Predicted fractions of patients without diarrhea (Grade ≥3) at specific, constant average cabozantinib concentrations


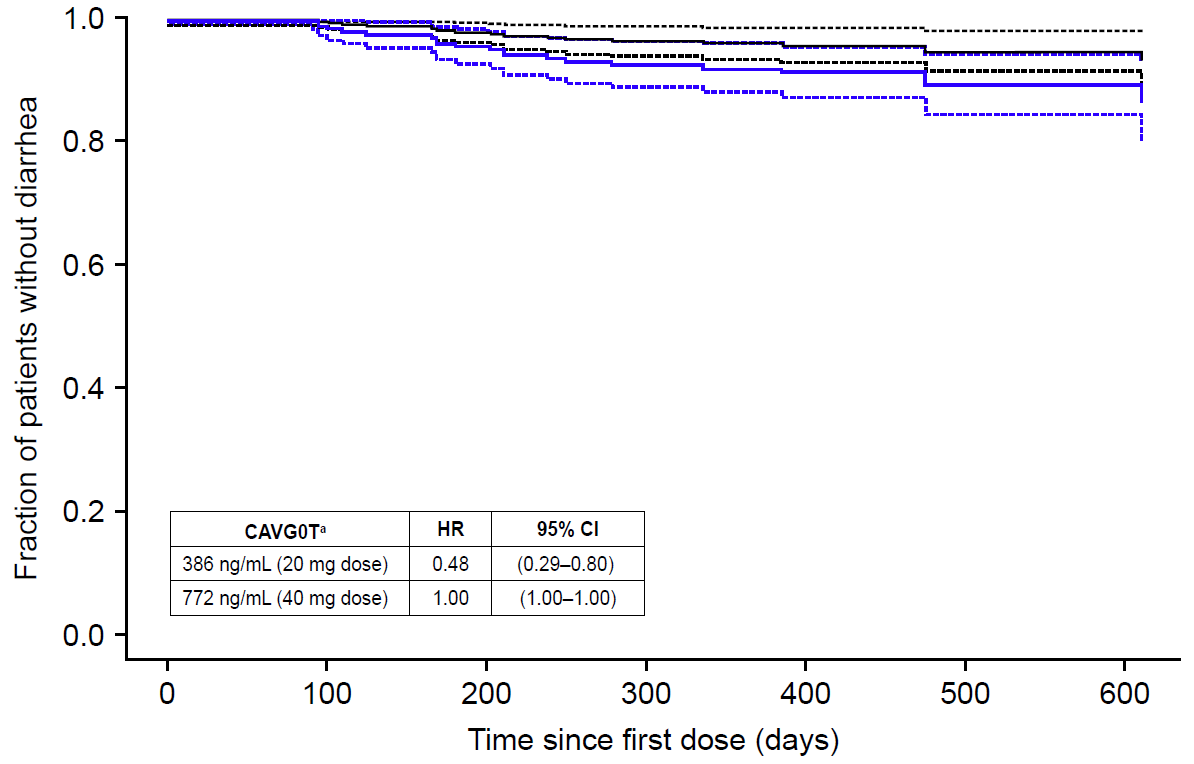


Typical individual predicted steady-state average cabozantinib concentration for the 20 mg (black) and 40 mg (blue, reference CAVG0T) doses are 386 and 772 ng/mL, respectively. The solid line represents the fraction of patients at each dose level without diarrhea over time. The dashed lines represent 95% confidence intervals

^a^Cabozantinib concentrations correspond to model predicted typical individual steady-state average concentrations for the 20 mg and 40 mg once daily dosing regimens

*CAVG0T* predicted average cabozantinib concentration from time zero to the event or censoring time, *CI* confidence interval, *HR* hazard ratio
